# Implementation of *MyChart for recruitment* at an academic medical center

**DOI:** 10.1017/cts.2024.605

**Published:** 2024-10-14

**Authors:** Carrie Dykes, Cody Gardner, Jack Chang, David Pinto, Karen Wilson, Martin S. Zand, Ann Dozier

**Affiliations:** 1 Clinical and Translational Science Institute, School of Medicine and Dentistry, University of Rochester, Rochester, NY, USA; 2 Department of Pediatrics, School of Medicine and Dentistry, University of Rochester, Rochester, NY, USA; 3 Department of Medicine, Division of Nephrology, School of Medicine and Dentistry, University of Rochester, Rochester, NY, USA; 4 Department of Public Health Sciences, School of Medicine and Dentistry, University of Rochester, Rochester, NY, USA

**Keywords:** Recruitment, electronic health record, electronic medical record, patient portal, response rate, translational science barrier

## Abstract

**Introduction:**

Recruitment of participants into research studies remains a major concern for investigators. Using clinical teams to identify potentially eligible patients can present a significant barrier. To overcome this, we implemented a process for using our patient portal, called MyChart, as a new institutional recruitment option utilizing our electronic health record’s existing functionality.

**Methods:**

To streamline the institutional approval process, we established a working group comprised of representatives from human subject protection, information technology, and privacy and vetted our process with many stakeholder groups. Our specific process for study approval is described and started with a consultation with our recruitment and retention function funded through our Clinical and Translational Science Award.

**Results:**

The time from consultation to the first message(s) sent ranged from 84 to 442 days and declined slightly over time. The overall patient response rate to MyChart messages about available research studies was 23% with one third of those saying they were interested in learning more. The response rate for Black and Hispanic patients was about 50% that of White patients.

**Conclusions:**

Many different types of studies from any medical specialty successfully identified interested patients using this option. Study teams needed support in defining appropriate inclusion/exclusion criteria to identify the relevant population in the electronic health records and they needed assistance writing study descriptions in plain language. Using MyChart for recruitment addressed a critical barrier and opened up the opportunity to provide a full recruitment consultation to identify additional recruitment channels the study teams would not have considered otherwise.

## Introduction

Recruitment of participants into clinical trials is a challenging barrier to conducting clinical trials [1]. Several different types of electronic communication used to inform potentially eligible research participants about study opportunities have been tried. These include social media advertisements, online websites with registries, text messaging, and patient portals [2–28]. The success of these recruitment channels varies depending on the research question and type of participants sought [18–33]. For example, recruitment using patient portals has been demonstrated for surgical patients [18], bariatric patients [32], and pediatric patients [19], to name a few. It has also been used to screen primary care patients [22] and to minimize bias in patient sampling [21].

The University of Rochester Medical Center (URMC) is an opt-in institution with a no “cold-calling” policy (patients cannot be contacted directly by researchers about a research study opportunity). Use of clinicians is a critical recruitment channel as patients have reported that they prefer to hear about studies from their providers [34]. However, patients are in favor of using the electronic medical record to learn directly about study opportunities [35]. At URMC, clinicians can only contact their patients (e.g. for a research study) but cannot contact patients cared for by other clinicians. This creates a recruitment barrier because clinicians often do not know what study opportunities are available or what their patients may qualify for. In addition, clinicians often lack time to recruit potentially eligible patients or provide permission to a researcher to contact their patient. Other potential barriers include a clinician’s view that they decide which studies a patient should participate in, mistrust of research or they do not want others to have access to “their” patients [36].

The URMC electronic medical record system, named eRecord, is EPIC®-based. URMC has 2.33 million patient records and 67% have active MyChart accounts. With the upgrade to Version May21 (October 2021), patients were provided their own “Research Studies” page as a part of their patient portal, called MyChart in the URMC eRecord system. This upgrade provided a new direct-to-patient recruitment channel. To date, there are no reports of how to implement this type of patient portal recruitment method at the enterprise level for all studies across an institution nor are there reports of the response rates. We implemented the use of MyChart for recruitment at URMC and offered this tool to all research teams recruiting patients. What follows describes our development of the process, the institutional approval, workflow, and our experience from January 2022 to January of 2023 including utilization, response and enrollment rates.

## Development of the stakeholder group/team and institutional approval process

In June 2020, the institution’s Information Systems Division shared that the upgrade to version May21 would include the Research Studies page. Thus, URMC patients who use MyChart would be able to opt in or out of research contact via MyChart, see what studies they are enrolled in and express interest in participating in additional/other study opportunities.

**Figure 1.**
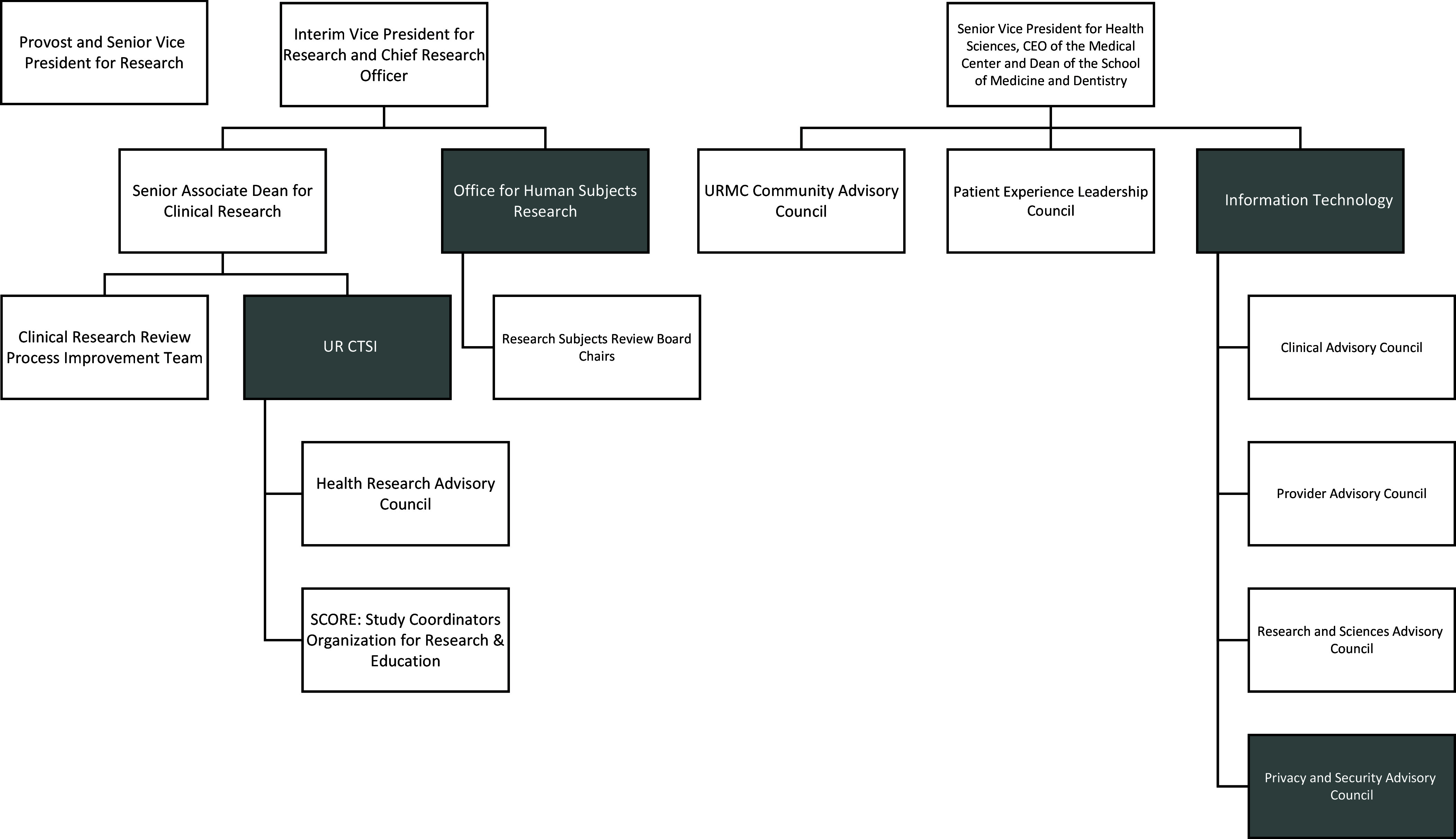
Organizational structure of the stakeholder groups involved in the development, review, and approval of the (MyChart for recruitment) MCfR process. The offices shown were represented in the working group. The offices in white reviewed and approved the process developed by the working group. CEO = chief executive officer; URMC = University of Rochester Medical Center; UR CTSI = University of Rochester Clinical and Translational Science Institute; MCfR = MyChart for Recruitment.

Key stakeholders were convened to establish how the Research Study pages and MyChart for Recruitment (MCfR) would be implemented, how patients would be informed of the change and how study teams could use it for recruitment. Stakeholders represented included: the Office of Human Subject Protection, the Information Systems Division (MyChart team), Academic and Research IT, the University of Rochester Clinical and Translational Science Institute (UR CTSI) Recruitment Unit, UR CTSI Informatics and the Office of Privacy and Security (Figure 1). Research faculty representatives were also included and the UR CTSI provided project implementation support. The group met monthly. After the working group developed a process for using MyChart to recruit patients (MyChart for Recruitment-MCfR), the process was presented to other stakeholder groups for feedback and approval (Figure 1).

Once approved by URMC and University Senior Leadership, the process was piloted with seven studies from May 2021 to September 2021. Experience with the seven pilot studies, including opt-out rates and patient feedback, informed refinements to our process.

In January 2022, MCfR was made available to all research teams in the medical center. All studies were approved with several exceptions. First, studies of a sensitive nature (e.g. interpersonal violence; substance use disorder) were excluded due to the possibility of negative impacts on patients if someone else saw the email. Second, we excluded studies that recruited individuals between the ages of 12 and17. These individuals, in addition to their parent/guardian, receive emails from their MyChart account, a MCfR email would be in violation of our institutional policy that prevents the direct recruitment of people age 12–17 into research studies. As a result, studies enrolling children age 0–11 or adults 18 and older were approved to use the tool. Lastly, due to concerns about providing additional individuals with access to patient data, only medical center based principal investigators were approved for MCfR use. This eliminated researchers from other Schools at the University of Rochester (e.g. Education, Arts, and Sciences).

## Patient communication plan

Starting in October of 2021, we established seven modes of communication to inform patients about MCfR including how they can learn more and how they can opt-out of receiving MyChart research messages. The project, branded for patients as *Research Connections,* utilized all locations possible where a patient interactions with the system. As examples, statements about research were added to: the welcome email for new MyChart users, the MyChart Terms and Conditions website, MyChart log in screen, and the Research Studies page after patients logged in.

## MyChart for recruitment process

**Figure 2.**
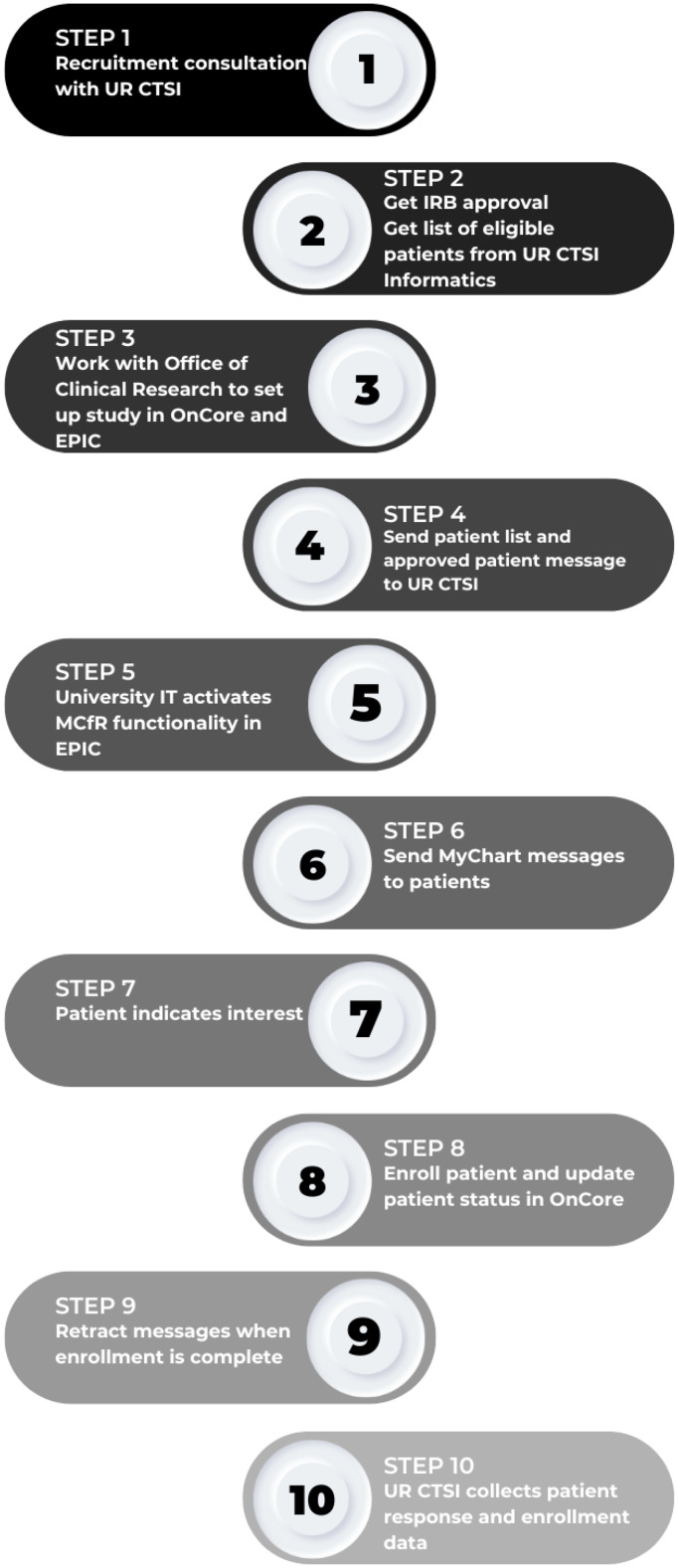
MyChart for recruitment process. UR CTSI = University of Rochester Clinical and Translational Science Institute; IRB = internal review board; University IT = University Information Technology.

Figure 2 shows the MyChart for Recruitment process.

### Step 1 – consultation

A consultation with the UR CTSI Recruitment Unit is required to determine if MCfR is an appropriate recruitment channel. This consultation also includes assistance with feasibility to determine if the protocol’s patient population exists within the URMC healthcare organization. We used the TriNetX, LLC Query Builder (TriNetX.com) from the TriNetX University of Rochester Network, which allows researchers to query electronic medical records (demographics, diagnoses, visits, procedures, medications, and laboratory tests) of all patients from the UR Medicine and get de-identified patient counts back. Additional recruitment channels are also reviewed as MCfR complements more traditional means of recruitment and study teams typically utilize multiple channels of recruitment.

### Step 2 – application

The study team submits their protocol for institutional review board (IRB) approval, indicating use of MCfR, what data elements from eRecord would be used to identify potentially eligible patients and the study description patients would see on their Research Studies page. All study descriptions are required to include the following language: *This study opportunity may not have been reviewed by your clinical care team. Click “yes, I’m interested” if you would like the study team to follow up with you about participating in this research study. Click “No, Thank You” if you would not like to be contacted about this study. If you no longer wish to receive email notifications about research studies through your MyChart Research Studies page, please click on the Do Not Contact button above. You may still receive regular MyChart messages from your clinical care team about research studies.* Once approved, teams submit a service request (clinical data query) to UR CTSI Informatics to generate a list of potentially eligible patients eligible.

### Steps 3–5 – eRecord study record

In order to use the functionality in eRecord, a study record is required. Following IRB approval, the study team and UR CTSI Office of Clinical Research enters the study into our clinical trial management system, OnCore, which is required to transfer study information over to eRecord. Both the list of potentially eligible patients and the IRB approved study description are then funneled through the UR CTSI to the MyChart team of URMC’s Information Systems Division where they are both loaded into the eRecord study record.

### Step 6 – messaging

Only a member of the study team with study coordinator security level access and trained to use eRecord can send MyChart messages. This role requires completion of relevant eRecord training. Teams also plan, based on their resources and study requirements, how many individuals to contact at any one time. The patient list provided to teams is intentionally limited to 2,000 patient contact messages at a time. This limits access to identifiable patient data. Although this is the default, the team can request more than one set of 2,000 patients. The lists are a random selection of patients or teams can request a subset based on demographic or medical criteria. The designated study coordinator sends potentially eligible patients a MyChart email or SMS text saying they have a message.

### Steps 7 and 8 – patient interest and enrollment

**Figure 3.**
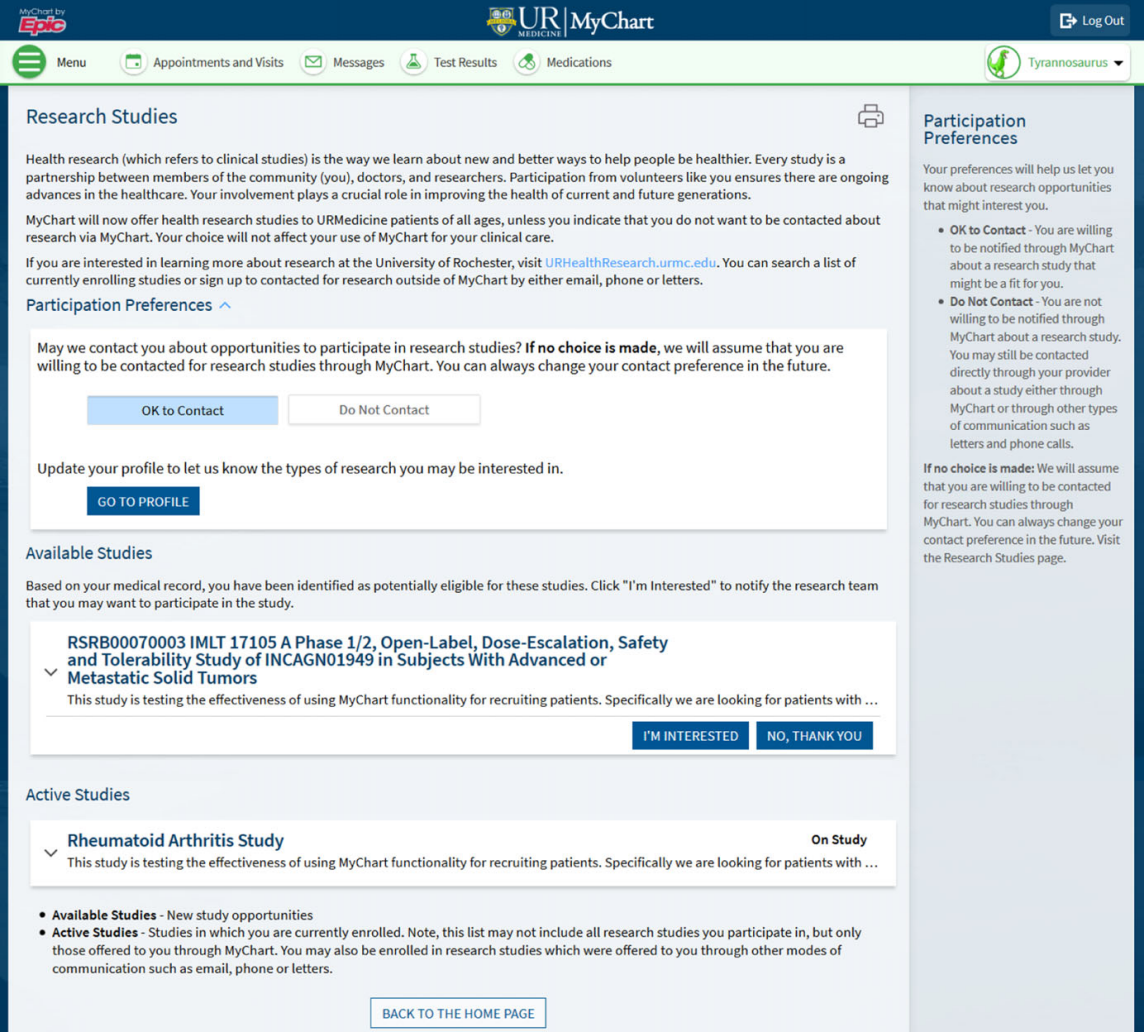
Screen capture from an example of a research studies page in the eRecord patient portal, MyChart. RSRB = research subjects review board.

Once in MyChart, the patient navigates to their research section with information about the study where they can see information about why they were contacted and about the specific study for which they are potentially eligible (Figure 3). The patient then selects “yes, I’m interested” (if interested) or “no, thank you” if they are not. In addition to indicating “not interested” in the study, patients also have the option to opt-out of future Research Connections communications about future research study opportunities. In eRecord’s Reporting Workbench, research team members can see who did or did not respond. Study teams are only allowed to send messages to the same patient twice. Patients can be contacted concurrently about more than one study. (Step 8) If patients select yes, the designated study coordinator receives a message in their eRecord in-basket. Per their study-specific protocol, the research team then follows up with interested patients and are encouraged to follow up within 2–3 business days. When patients consent, the study team enters the patient into the clinical trial management system (OnCore) and updates the patient’s status accordingly as they move through the study. This is a manual process, so the MyChart for Recruitment team reminds study teams every 6 months to enroll patients in OnCore and keep their status updated.

### Step 9 – retraction

Study opportunities remain on a patient’s Research Page until the patient responds or until they are retracted. The latter can occur if the study team determines the patient is no longer eligible or at the end of the recruitment period when the study coordinator removes each unanswered message one-by-one. All patients who expressed interest were contact by the study team prior enrollment closure. All patients who were successfully contacted after expressing interest were screened for the study.

### Step 10 – data collection

The eRecord MyChart team extracts data from the EMR that includes patient demographics, number of messages sent, number of patients who declined the invitation, number of patients who expressed interest and their study enrollment status.

## Data collection and evaluation

**Figure 4.**
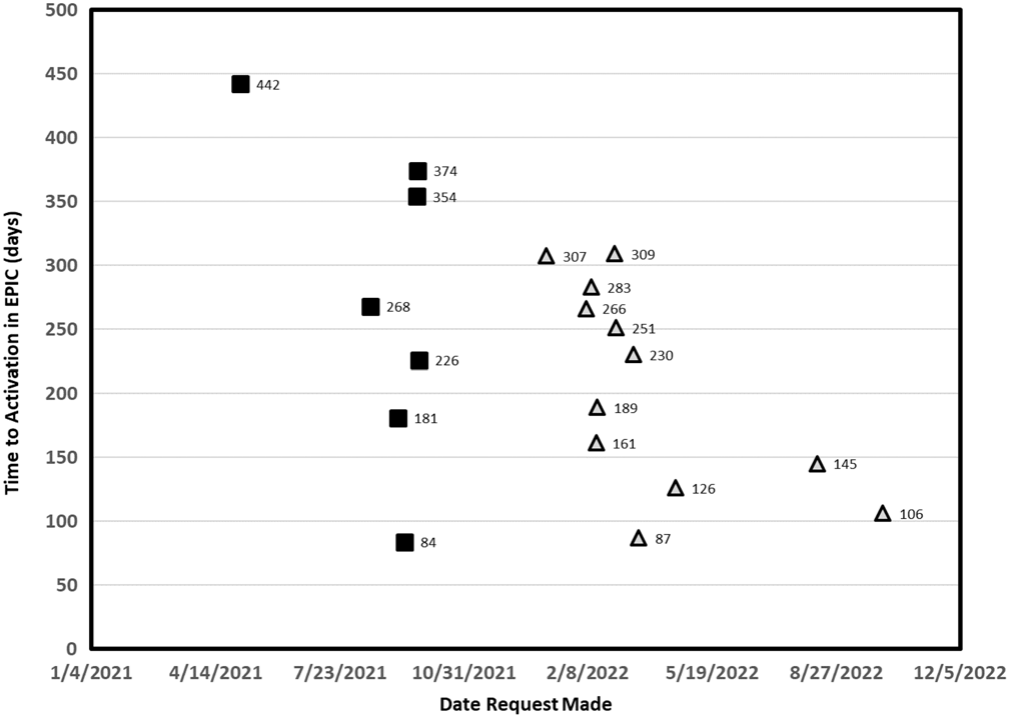
The *x*-axis is the date the study team requested to use MyChart for recruitment and the *y*-axis is the number of days from time of request to activation of MyChart functionality in eRecord. 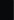 Studies that were part of the pilot phase. 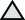 Study requests received after the pilot phase.

From 2021 through January 2023, we received requests to use MCfR from 59 studies and consulted on 51 (eight studies withdrew interest prior to consultation). Based on the 51 studies, the mean time to complete the MCfR process from consultation to sending messages was 231 ± 101 days (Figure 4). The average completion time decreased slightly over time. Nineteen studies completed set up for MyChart for Recruitment at the time of this data analysis. Thirty-two studies were still going through the set up process. On average getting a study activated to use MCfR took about 7 hours total spread out over time with another 2–3 hours to perform the EHR data query to identify the eligible patients.

From 2021 to January 2023, 19 studies sent 8,240 messages to 8,168 patients. Patients received messages from one to three studies. Table 1 shows the number of studies by participating departments that included both primary care (e.g. family medicine; obstetrics and gynecology) and specialty (e.g. psychiatry, surgery, neurology).

**Table 1.** Departments that sent messages and the number of studies from each department

Department	# of Studies
Dermatology	1
Dentistry	1
Family Medicine	1
Gastroenterology and Hepatology	1
Neurology	2
Neurosurgery	1
Obstetrics and Gynaecology	1
Oncology	2
Otolaryngology	1
Public Health Sciences	2
Psychiatry	4
Pulmonology	1
Surgery	1

The 1,878 responses to 8,240 messages represent an overall response rate of 23%. Of these responses, 1,242 (66%) patients declined interest and the remaining 636 (34%) expressed interested. Studies sent between 1 and 2,199 messages. One study only messaged one patient while other studies messaged hundreds or thousands depending on who was eligible. The study that messaged one patient did not get a response and a second study that messaged five patients received a response from all five. For studies that messaged more than 100 patients, the range of response rates by study was 7%–37% with a median of 20% and a mean of 22%. Five percent of patients opted out of receiving future messages.

Table 2 shows the demographic characteristics of all non-deceased patients in the EMR and the patients who received a message and their respective response rates (sum of declined and interested). There were more women contacted compared to men and women had a higher response rate. There were fewer younger people and children contacted and their response rates were lower compared to people 35–95 years of age. Reflecting our patient population, there were more White patients contacted, and they had double the response rate of individuals who were Hispanic or from other races. Compared to all patients in the EMR, there were more people age 35–64 contacted. There were also fewer patients contacted with unknown ethnicity or race.

**Table 2.** Demographics of patients receiving MyChart messages and response rates^e^

		All Patients^d^	Patients Receiving Study Opportunity	
		#^f^ of Patients (%)	# of Patients (%)	Response Rate (%)
All Patients^b^		2,120,116 (100)	8,168 (100)	23
Sex^a^	Female	1,114,389 (53)	4,713 (57)	24
	Male	1,004,332 (47)	3,526 (43)	20
	Sex unknown	1,395 (0.1)	0 (0)	
Age^c^	0–10	184,011 (9)	16 (0.2)	0
	11–17	154,192 (7)	0 (0)	
	18–34	483,426 (23)	1,067 (12.9)	19
	35–54	510,628 (24)	2,610 (31.7)	22
	55–64	272,042 (13)	2,253 (27.3)	24
	65–95	515,817 (24)	2,292 (27.8)	25
Ethnicity	Hispanic	96,800 (5%)	361 (4)	14
	Non-Hispanic	1,313,719 (62%)	6,999 (85)	24
	Unknown	709,597 (33%)	880 (11)	20
Race	American Indian or Alaska Native	3,258 (0.2)	28 (0.3)	11
	Asian	44,390 (2)	604 (7.3)	9
	Black or African American	187,942 (9)	717 (8.7)	14
	White	1,405,242 (66)	6,564 (79.7)	26
	Unknown	447,800 (21)	327 (4.0)	12

a Sex was missing for one patient.

b Eighty patients were deceased at the time the data were pulled, but not necessarily at the time messages were sent.

c Age for two patients was missing.

d Multiracial patients were not included in the All Patients column. They represented 1% of the total population.

e Multiracial patients were listed under the racial category listed first in their record.

f # = Number.

Table 3 depicts the study status of the 636 “interested” patients as of January 2023, based on data provided by participating study teams. The 480 “interested” patients were either not yet contacted by the study team or the study team had not updated the enrollment status in the clinical trial management system (OnCore). Sixteen patients were interested, but not eligible, while seven patients were eligible and but not yet on study. Fourteen patients were waiting to be consented, while 47 patients had been enrolled and completed the study (off study). Overall, 25% of interested patients moved forward through the study process.

**Table 3.** Study status of interested patients (*N* = 636)

Study Status^a^	# of patients (%)
Interested	480 (75)
Eligible	7 (1)
Not eligible	16 (3)
Off study	47 (7)
On study	67 (11)
On treatment	5 (0.8)
Waiting for consent	14 (2)

a As of 01/25/2023.

### Lessons learned

Many different types of studies can benefit from using MCfR to contact more potential participants than they might otherwise have been able to access. This includes not only interventional studies for specific conditions, but observational and registry studies. Studies could have very specific inclusion/exclusion criteria or very broad criteria. During the consultations, we found that investigators needed assistance defining the best inclusion and exclusion criteria to find the optimal population to query. Investigators also needed additional support writing study descriptions in plain language. Often teams would use scientific language or would use other materials they created for recruitment such as language on flyers, emails, or phone scripts. We provided rewrites of study descriptions so the content explained the importance and expectations of the study in a clear and concise manner.

With the launch of MCfR, there was confusion on the part of investigators about the policy for the use of MyChart for research. Our institutional policy states that MyChart can only be used for recruitment, specifically, for sending messages introducing a potential study. The only exception to this is that a clinician can send a regular direct MyChart message about a potential study to their own patients. However, how a clinician claims a patient as their own needed clarification. We determined that the clinician needed to be the assigned primary provider in eRecord and could not claim a patient as theirs if they only see them when other clinicians were not available. In addition, education about the MyChart policy needed to include information indicating that MyChart could only be used for only initial contact and no other downstream research communications.

Our results to date show that 75% of patients remain with an “interested” status in eRecord and have not proceeded to the next step of eligibility. This could be due to the study team not contacting patients who are interested, not being able to get a hold of interested patients, or not updating the patient enrollment status in OnCore. Therefore, study teams may need to be reminded to update statuses or provided with other tools and resources that help them reach patients after they express interest. This may also require more guidance for study teams on how to manage the number of messages they send out at any one time to assure that interested individuals are contacted in a timely manner.

In order to limit access to patient data as well as help teams manage their workload, we could implement a test run of fewer than 2000 patients. Based on the response rate and ability to enroll the test population of patients, study teams could then adjust the number of patients to message.

### Strengths

First, in developing MCfR, collaboration across multiple stakeholders reduced downstream implementation barriers. Second, our recruitment consultations increased substantially. Through this tool study teams were provided with information about other approaches to strengthen their recruitment that they might not otherwise have considered. Third, this new channel addressed a critical barrier to accessing patients for recruitment into research studies. Patients who might not have otherwise been contacted are now provided access to study opportunities. Lastly, this provided data on patient interest in participation in research studies. Our response rate of 23% was higher than previously published response rates to patient portal messages, which ranged from 1.7%–7.0% [21,23,25,31,37].

### Limitations

This approach had its limitations. As noted earlier, not all interested investigators were eligible. The process takes a number of months and some teams may need a quicker turnaround. Study teams may not appreciate the bandwidth to respond to inquiries. While we are exploring ways to include 12–17-year-old patients into MCfR, we have thus far been limited by the constraints of parental consent and adolescent access to confidential care. In addition, we had fewer patients contacted from traditionally underserved populations making it difficult to evaluate this recruitment method in those patients. There were differences in the demographics of patients who received a study opportunity when compared to all patients in the EMR. This could be due to the types of studies that used this method as well as the choice study teams had in who they wanted to contact. Study teams were allowed to prioritize contact of eligible patients based on demographics.

## Conclusions

This recruitment option generated significant interest across multiple departments and is viewed as an important new tool for direct recruitment of patients. It also created greater visibility of other recruitment tools. Additional refinements to address the turnaround and response issues will further strengthen the utility of this approach.
